# Heartbeats in Distress: Unveiling Cardiac Sarcoidosis Through Palpitations

**DOI:** 10.7759/cureus.52833

**Published:** 2024-01-23

**Authors:** Temitope A Tobun, Ndausung Udongwo, Joshua Stephens, Joseph Heaton, Farah Bashir, Theodora Esomonye, Mohammed Alkubeysi, Jalal Ghali

**Affiliations:** 1 Internal Medicine, Morehouse School of Medicine, Atlanta, USA; 2 Cardiology, Morehouse School of Medicine, Atlanta, USA; 3 Internal Medicine, Jersey Shore University Medical Center, New Jersey, USA

**Keywords:** sarcoidosis, palpitations, cardiac sarcoidosis, positron emission tomography with 2-deoxy-2-[fluorine-18]fluoro-d-glucose, cardiovascular magnetic resonance imaging

## Abstract

Cardiac sarcoidosis (CS), a rare complication of systemic sarcoidosis, can have subtle or no symptoms. It is characterized by granuloma formation in the myocardium, which can occur in isolation or alongside systemic sarcoidosis. Clinical manifestations include conduction system disorders (e.g., atrioventricular block and ventricular tachyarrhythmia), heart failure, and sudden cardiac death. Timely evaluation and screening for CS are crucial, especially in systemic sarcoidosis patients with limited symptoms. We present the case of a 50-year-old African-American male diagnosed with cardiac sarcoidosis following a recent diagnosis of pulmonary sarcoidosis after experiencing tachycardia for two years, as confirmed by imaging studies.

## Introduction

Sarcoidosis is a systemic granulomatous disease that can affect multiple organs, with prevalence varying by region [1]. In the United States, the prevalence is estimated at approximately 60 per 100,000 adults [1,2]. Notably, the incidence and prevalence are higher in African Americans than in other racial groups [2]. Cardiac sarcoidosis (CS) is a relatively rare complication, diagnosed in 2-5% of individuals with pulmonary or systemic sarcoidosis [3]. However, it may be more common than recognized, affecting up to 30% of Americans with sarcoidosis, with higher rates observed in Japan [3]. It can present with a range of cardiac issues, including heart failure, arrhythmias, conduction abnormalities, and sudden cardiac death, often making it challenging to diagnose in clinical practice [4]. Diagnosing CS involves a combination of imaging techniques like electrocardiogram, transthoracic echocardiogram, cardiac MRI, fluorodeoxyglucose (FDG)-positron emission tomography (PET), and biopsy. Electrophysiology studies, FDG-PET, or cardiac magnetic resonance (CMR)-guided endomyocardial biopsy can enhance diagnostic sensitivity to approximately 50% [5]. In this case, we present a 50-year-old African-American male recently diagnosed with pulmonary sarcoidosis. He had a two-year history of tachycardia, initially attributed to post-COVID syndrome. However, cardiovascular magnetic resonance imaging revealed findings consistent with CS. 

## Case presentation

A 50-year-old male with a past medical history of type II diabetes, hypertension, obesity, systemic sarcoidosis, and recently diagnosed pulmonary sarcoidosis presented to the clinic with complaints of palpitation, occasional shortness of breath, paroxysmal nocturnal dyspnea, and dyspnea on exertion. He reports that palpitations were initially attributed to post-COVID palpitations in 2020. Previous electrocardiograms (ECG) have only shown sinus tachycardia. He endorsed an extensive family history of coronary artery disease (father, brother, and sister) but no family history of sarcoidosis. 

**Figure 1:**
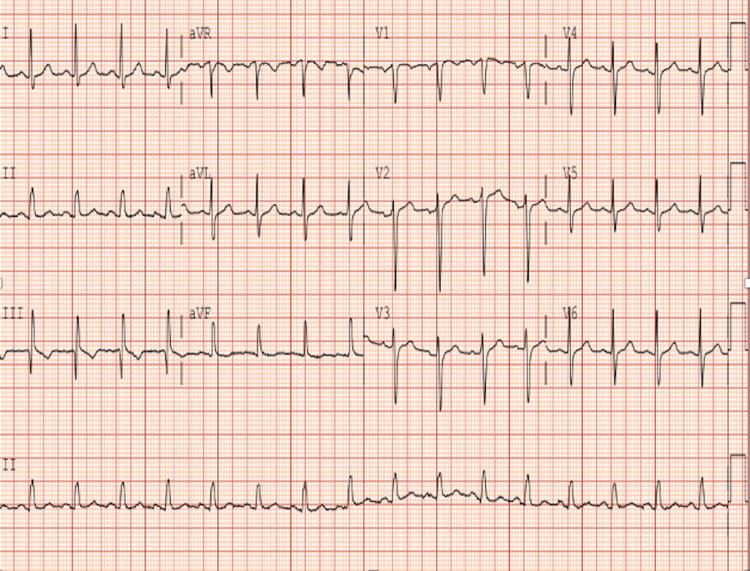
Electrocardiogram Normal sinus rhythm, rate of 96, with no ST/T wave changes.

On presentation, his initial vitals were as follows: blood pressure of 129/87 mmHg, heart rate of 106 beats per minute, respiratory rate of 20 breaths per minute, and oxygen saturation of 96% on ambient air. Home medications were candesartan 100 mg, amlodipine 10 mg, metoprolol succinate 100 mg, metformin 500 mg, all taken daily. On physical examination, he was not in any acute distress, and his cardiovascular and pulmonary examination was unremarkable. Initial laboratory findings were unremarkable, as shown in Table 1. ECG revealed a sinus rhythm, rate of 96 beats per minute, normal axis, with no ST or T wave changes (Figure 1). 

**Table 1:** Initial laboratory findings

Serum	Results	Reference range
White blood cells (K/mcL)	5.9	3.8 - 10.7
Hemoglobin (gm/dL)	13.6	13.2 - 17.7
Mean corpuscular volume (fL)	72	81 - 100.2
Platelet count (K/mcL)	320	148 - 362
Glucose (mg/dL)	93	70 - 125
Blood urea nitrogen (mg/dL)	18	8 - 22
Creatinine (mg/dL)	1.0	0.7 - 1.2
Sodium (meq/L)	142	132 - 144
Potassium (meq/L)	3.7	3.4 - 5.1
Chloride (meq/L)	105	101 - 111
Calcium (mg/dL)	8.9	8.9 - 10.3
Magnesium (mg/dL)	1.9	1.5 - 2.6
Bicarbonate (meq/L)	26	22 - 32
Alkaline phosphatase (U/L)	68	38 - 126
Total protein (gm/dL)	8.2	6.0 - 8.3
Albumin (gm/dL)	3.9	3.5 - 5.0
Total bilirubin (mg/dL)	0.6	0.3 - 1.6
Aspartate aminotransferase (U/L)	25	10 - 42
Alanine aminotransferase (U/L)	34	17 - 63
Troponin (ng/L)	4	<20

**Figure 2:**
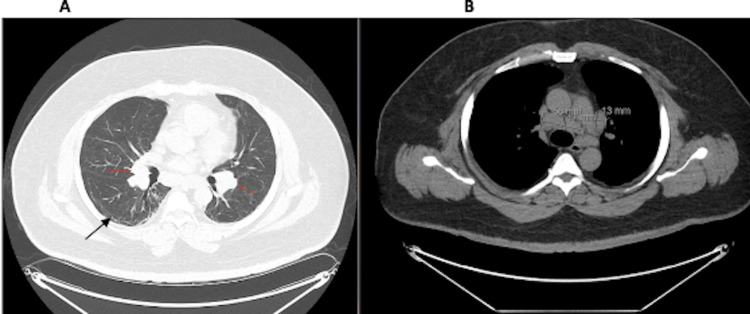
Computed tomography scan of the chest Pulmonary fibrosis (black arrow in image A) and mediastinal lymphadenopathy (red arrows in image A and measured lymph nodes in image B)

A computed tomography (CT) scan of the chest showed unchanged pulmonary fibrotic changes from alveolar damage secondary to prior COVID-19 infection and increased mediastinal lymphadenopathy (Figures 2A-B).

**Figure 3:**
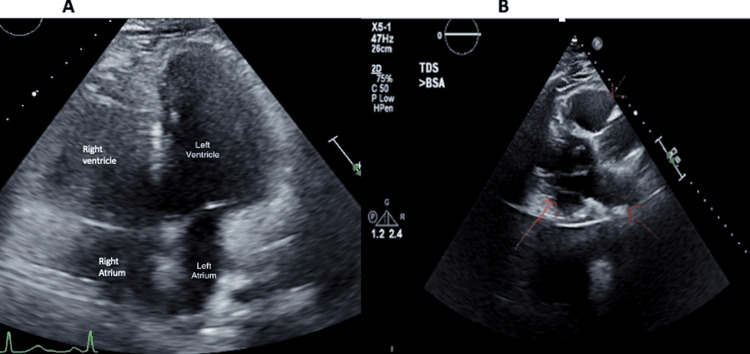
Transthoracic echocardiogram Mild concentric left ventricular hypertrophy with ejection fraction of 55-60%, mildly dilated left atrium, and right ventricle (labeled chambers in image A and red arrows in image B).

Bronchoscopy with fine needle aspiration revealed a generous cellblock that showed non-necrotizing granulomata in a background of lymphoid tissues but no malignancy. Transthoracic echocardiogram showed a mild concentric left ventricular hypertrophy (LVH) with an ejection fraction (EF) of 55-60% and a mildly dilated left atrium and right ventricle (Figures 3A-B). 

**Figure 4:**
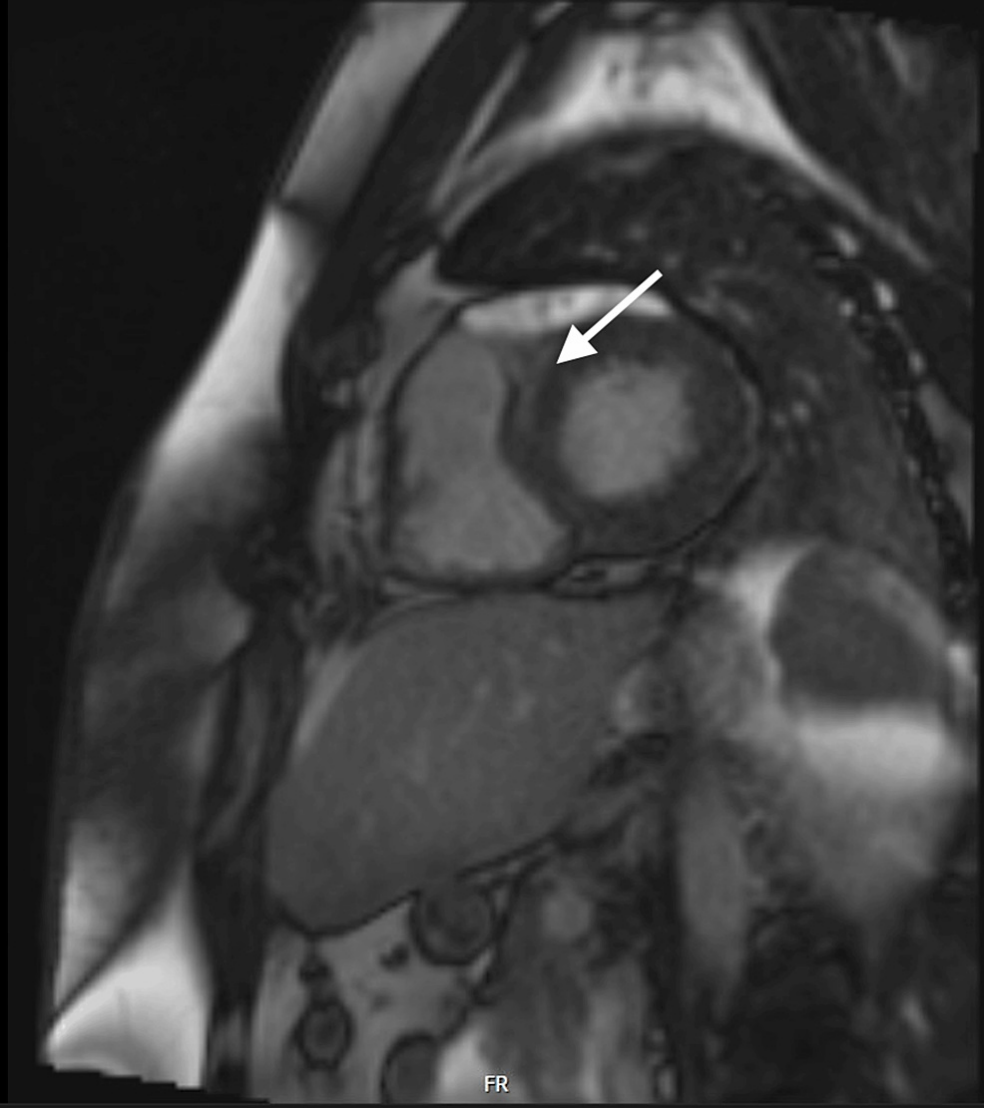
Cardiac MRI Late gadolinium enhancement (LGE) at the basal antero-septal segment (white arrow).

The CMR imaging revealed severe concentric LVH with EF of 57%, mid myocardial late gadolinium enhancement (LGE) suggesting a non-ischemic myocardial fibrosis/scar at the basal antero-septal segment, bilateral hilar and mediastinal lymphadenopathy, and mild to moderate tricuspid regurgitation (Figure 4). 

**Figure 5:**
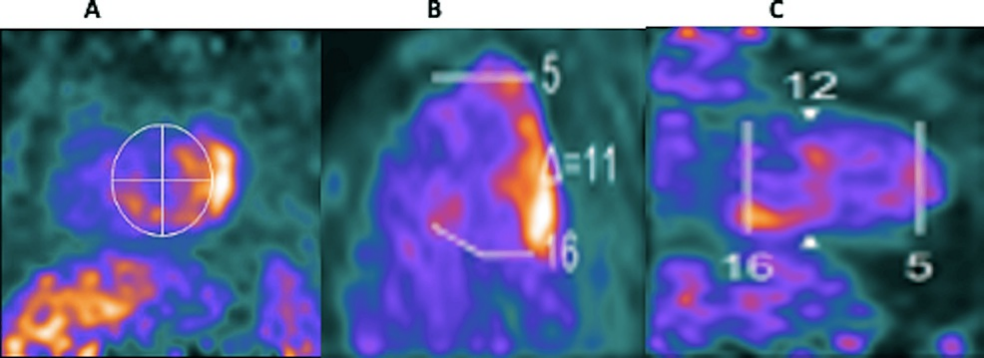
Positron emission tomography with 2-deoxy-2-[fluorine-18]fluoro-D-glucose (FDG-PET) Uptake in the basal inferior (A), inferolateral (B), and inferoseptal (C) walls suspicious for active sarcoidosis.

Positron emission tomography with 2-deoxy-2-[fluorine-18]fluoro-D-glucose (FDG-PET) showed an increased FDG uptake in the basal inferior, inferolateral, and inferoseptal walls suspicious for active sarcoidosis (Figures 5A-C). 

Our patient's clinical symptoms and imaging were consistent with cardiac sarcoidosis. A 14-day Holter monitor was ordered to rule out conductive tissue abnormalities. He was started on prednisone 20 mg by pulmonology prior to the clinic visit due to the recent diagnosis of pulmonary sarcoidosis. He was discharged in stable condition and has been regularly following up with cardiology and pulmonology. He is currently on prednisone 10 mg daily with a resolution of tachycardia. 

## Discussion

Cardiac sarcoidosis affects all three layers of the heart, mainly the myocardium, and is a severe complication of systemic sarcoidosis [6]. Myocardial granulomas were found in 27% of pulmonary sarcoidosis autopsies, according to Silverman et al. [7]. Cardiac involvement in patients with sarcoidosis is being increasingly recognized [8]. Despite these findings, only 5% of patients with sarcoidosis have clinical manifestations of cardiac disease. Only 40-50% of patients with cardiac sarcoidosis at autopsy have the correct diagnosis made during their lifetime [8]. It has been observed that a considerable proportion, specifically 37%, of sarcoidosis patients with cardiac involvement seem to be asymptomatic, indicating a possible lack of clinical signs or symptoms of the disease [9]. Clinical manifestation of CS includes conduction system disorder like atrioventricular (AV) block, ventricular tachyarrhythmia, heart failure, and sudden cardiac death. Table 2 below shows other clinical manifestations and prevalence of CS [10].

**Table 2:** Clinical manifestations and prevalence of cardiac sarcoidosis Source: [10]

Clinical manifestations	Reported prevalence
Arrhythmias	
Atrioventricular block	26-62%
Bundle branch block	12-61%
Supraventricular tachycardia	0-15%
Ventricular tachycardia	2-42%
Sudden cardiac death	12-65%
Cardiomyopathy	
Congestive heart failure (left ventricular systolic failure, heart failure with preserved ejection fraction or restrictive disease, and right ventricular failure secondary to pulmonary disease)	10-30%
Pericardial manifestations	
Pericardial effusion (common) and pericarditis (rare)	20%

A detailed medical history, physical examination, echocardiography (ECHO), and other imaging techniques are required for a precise and early diagnosis to prevent developing life-threatening complications [6]. Imaging techniques include CMR, 18F-FDG PET, and electrophysiology studies. ECHO findings of CS include thinning of the basal intraventricular septum or left ventricular (LV) free wall. Other findings of CS may mimic coronary artery disease (LV regional wall motion abnormalities or apical aneurysm) or diastolic dysfunction [11]. Further investigation should be prompted by subtle abnormalities in diastolic flow patterns in a patient with extracardiac sarcoidosis [10]. One study found that 14% of patients with pulmonary sarcoidosis had diastolic dysfunction ultimately attributed to CS [12]. On T2-weighted images, increased signal intensity may be observed on a CMR scan, indicating edema in acute myocardial inflammation. Early gadolinium-enhanced images may also show an increased signal intensity. In the case of fibro-granulomatous scarring, late gadolinium enhancement is usually found in the mid-myocardium and epicardium, unlike ischemic disease, where it predominantly appears in the endocardium. The basal and lateral segments of the left ventricle and the papillary muscles are the areas where fibro-granulomatous scarring is more common [10].

Ishimaru et al. performed a study where 18F-FDG PET was used to investigate CS. They categorized the images into four patterns: none, diffuse, focal, and focal on diffuse. Patients exhibited all four patterns, with a higher prevalence of focal and focal on diffuse patterns [13]. In another study, 18F-FDG PET uptake was frequently observed in the basal and mid-anteroseptal-lateral walls of the left ventricle [14]. Vita et al. reviewed the use of evaluating CS alone with CMR or PET alone versus a combination of both imaging in 107 patients and found that a combination of both imaging provides complementary value for estimating the likelihood of CS among patients with suspected CS [15]. It's worth noting that CMR is particularly adept at identifying fibrotic changes in the nonviable myocardium. However, it may not be as effective in detecting early inflammatory changes during the early stages of cardiac sarcoidosis. In such cases, 18F-FDG PET may prove to be a more effective diagnostic tool [16]. 

Corticosteroids are used as the first-line therapy of CS and may halt the progression of the disease [17]. Steroid-sparing immunosuppressive agents, including methotrexate, cyclophosphamide, azathioprine, rituximab, and tumor necrosis factor (TNF) inhibitors, are used as second-third-line therapy or with steroids [18]. The duration of treatment is dependent on the response to the initial therapy. Typically, patients are re-evaluated two to three months after initiation of steroid therapy to determine disease activity and need to taper doses of medication, but the effective duration of treatment remains unclear. The prognosis of CS remains ill-defined regardless of immunosuppressive therapy. Yazaki et al. performed a study in Japanese patients, showing that starting corticosteroids before systolic dysfunction resulted in an excellent clinical outcome. Still, the severity of heart failure is a critical factor that strongly predicts mortality [19].

## Conclusions

Cardiac sarcoidosis is a rare manifestation of systemic sarcoidosis; a high index of clinical suspicion is warranted when a patient presents with non-specific cardiac symptoms. Prompt diagnosis is essential due to poor prognosis. Symptoms may overlap with other medical disease like myocarditis. Clinical and imaging findings will exclude alternative diagnoses, including cardiac MRI, fluorodeoxyglucose-positron emission tomography, and electrophysiology study. 
